# 1274. Activity of Ceftolozane/Tazobactam, Imipenem/Relebactam, and Comparators Against *Pseudomonas aeruginosa* Isolates from Patients with Bloodstream and Other Infection Types—SMART United States/Canada 2018-2019

**DOI:** 10.1093/ofid/ofab466.1466

**Published:** 2021-12-04

**Authors:** Sibylle Lob, Meredith Hackel, C Andrew DeRyke, Kelly Harris, Katherine Young, Mary Motyl, Daniel F Sahm

**Affiliations:** 1 IHMA, Inc., Schaumburg, IL; 2 Merck & Co., Inc., Kenilworth, New Jersey; 3 Merck & Co. Inc, Kenilworth, New Jersey; 4 Merck & Co, Inc, Kenilworth, NJ

## Abstract

**Background:**

Ceftolozane/tazobactam (C/T), an antipseudomonal cephalosporin combined with a β-lactamase inhibitor, was approved for treatment of complicated urinary tract (cUTI) and intraabdominal infections (cIAI), and hospital-acquired/ventilator-associated bacterial pneumonia (HAP/VAP). Imipenem/relebactam (IMI/REL) is a combination of imipenem/cilastatin with relebactam, an inhibitor of class A and C β-lactamases. IMI/REL was approved for HAP/VAP and for infections due to aerobic gram-negative organisms in adults with limited treatment options (e.g., cUTI, cIAI). We compared the activity of C/T and IMI/REL against *P. aeruginosa* from bloodstream infections (BSI) to those from other infection types.

**Methods:**

As part of the SMART program, 24 hospitals in the US and 8 in Canada each collected up to 250 consecutive gram-negative isolates per year in 2018-2019 from patients with BSI, lower respiratory tract infections (LRTI), IAI, and UTI. A total of 2351 *Pa* isolates were collected. MICs were determined using CLSI broth microdilution and breakpoints.

**Results:**

*Pa* isolates from BSI tended to show higher susceptibility than IAI, UTI, and especially LRTI isolates (Table). Susceptibility to the tested comparator β-lactams was 11-12 percentage points lower among LRTI than BSI isolates, while C/T and IMI/REL susceptibility was only 2-5% lower. Even among BSI isolates, the comparator β-lactams were active against only 75-88% of isolates, while C/T and IMI/REL were active against >95%. Only amikacin showed higher activity. Analyzing coverage by either C/T or IMI/REL, 98.7% of *Pa* isolates from BSI were susceptible to one or both agents. C/T and IMI/REL maintained activity against 89% and 69% of meropenem-nonsusceptible (MEM-NS) *Pa* isolates from BSI (n=36), respectively, and 87% and 76% of piperacillin/tazobactam (P/T)-NS *Pa* (n=38).

Results Table

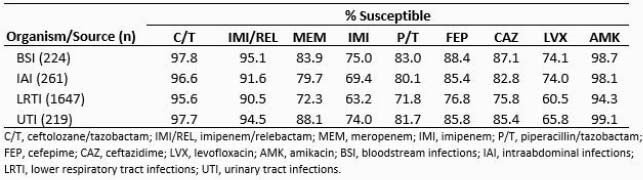

**Conclusion:**

Even among BSI isolates, which were generally more susceptible than those from other infection types, *Pa* susceptibility to commonly used β-lactams like MEM and P/T was < 90%, 7-23% lower than C/T and IMI/REL. Given the desirability of β-lactams among clinicians and the >98% coverage by either C/T or IMI/REL of *Pa* isolates from BSI, both agents represent important options in the treatment of patients with BSI.

**Disclosures:**

**Sibylle Lob, PhD**, **IHMA** (Employee)**Pfizer, Inc.** (Independent Contractor) **Meredith Hackel, PhD MPH**, **IHMA** (Employee)**Pfizer, Inc.** (Independent Contractor) **C. Andrew DeRyke, PharmD**, **Merck & Co., Inc.** (Employee, Shareholder) **Kelly Harris, PharmD, BCPS**, **Merck & Co. Inc** (Employee) **Katherine Young, MS**, **Merck** (Employee) **Mary Motyl, PhD**, **Merck & Co., Inc.** (Employee, Shareholder) **Daniel F. Sahm, PhD**, **IHMA** (Employee)**Pfizer, Inc.** (Independent Contractor)

